# Blinded predictions of distribution coefficients in the SAMPL5 challenge

**DOI:** 10.1007/s10822-016-9969-1

**Published:** 2016-09-27

**Authors:** Stefano Bosisio, Antonia S. J. S. Mey, Julien Michel

**Affiliations:** EaStCHEM School of Chemistry, University of Edinburgh, David Brewster Road, Edinburgh, EH9 3FJ UK

**Keywords:** SAMPL5, Distribution coefficient, $\log \hbox {D}$

## Abstract

In the context of the SAMPL5 challenge water-cyclohexane distribution coefficients for 53 drug-like molecules were predicted. Four different models based on molecular dynamics free energy calculations were tested. All models initially assumed only one chemical state present in aqueous or organic phases. *Model A* is based on results from an alchemical annihilation scheme; *model B* adds a long range correction for the Lennard Jones potentials to *model A*; *model C* adds charging free energy corrections; *model D* applies the charging correction from *model C* to ionizable species only. *Model A* and *B* perform better in terms of mean-unsigned error ($\hbox {MUE}=6.79<6.87<6.95 \log$ D units − 95 % confidence interval) and determination coefficient $(\hbox {R}^2 = 0.26< 0.27< 0.28)$, while charging corrections lead to poorer results with *model D* ($\hbox {MUE}=12.8<12.63<12.98$ and $\hbox {R}^2 = 0.16<0.17<0.18$). Because overall errors were large, a retrospective analysis that allowed co-existence of ionisable and neutral species of a molecule in aqueous phase was investigated. This considerably reduced systematic errors ($\hbox {MUE}=1.87<1.97<2.07$ and $\hbox {R}^2 = 0.35<0.40<0.45$). Overall accurate $\log D$ predictions for drug-like molecules that may adopt multiple tautomers and charge states proved difficult, indicating a need for methodological advances to enable satisfactory treatment by explicit-solvent molecular simulations.

**Electronic supplementary material:**

The online version of this article (doi:10.1007/s10822-016-9969-1) contains supplementary material, which is available to authorized users.

## Introduction

To help assess the predictive power of computational methods for molecular modelling the Statistical Assessment of the Modeling of Proteins and Ligands (SAMPL) was created almost 10 years ago [1, 2]. In 2015 the $5\mathrm{th}$ SAMPL challenge was announced and comprised of two main objectives: the blinded prediction of binding affinities of a set of host-guest complexes and the prediction of distribution coefficients for a library of 53 drug-like molecules. Since there is significant interest in using molecular simulation methods to support structure-based design of ligands for biomolecules [3], reliable predictions of host-guest binding affinities and distribution coefficients of drug-like molecules are important. These systems serve as a stepping stones towards reliable molecular modelling of more challenging biomolecules. A companion article describes results from our group for host-guest binding affinity predictions [4], and this report describes our efforts to predict distribution coefficients for these 53 drug-like molecules using molecular simulation methods.

This is the first time since the start of the SAMPL challenges, that a blinded prediction of distribution coefficients was included in the challenge. Distribution coefficients are an important quantity in medicinal chemistry [5, 6] and their measurements give useful information on potential ADME properties of drug-like small molecule. Experimentally it is straightforward to measure partition coefficients, namely the logarithm of the ratio of the un-ionized species between an organic phase, e.g. octanol, and an aqueous phase, i.e water, calculated as [7–10]:

1 $$\log P = \log \frac{[A]_{\mathrm{o}}}{[A]_{\mathrm{w}}},$$

where $[A]_{\mathrm{o}}$ is the concentration of the solute in the organic phase, and $[A]_{\mathrm{w}}$ the concentration in the water phase. However, the partition coefficient neglects to account that different forms of molecule A may co-exist as a mixture of protomeric and tautomeric states. Taking this into consideration leads to definition of a distribution coefficient, $\log D$:

2 $$\log D = \log \left( \frac{[A]_{\mathrm{o}} + [A]^+_{\mathrm{o}}}{[A]_{\mathrm{w}} + [A]^+_{\mathrm{w}}} \right) ,$$

where $[A]_{\mathrm{o}}$ and $[A]^+_{\mathrm{o}}$ are the concentration of the neutral and protonated species (all possible protonation states) in the organic phase, while $[A]_{\mathrm{w}}$ and $[A]^+_{\mathrm{w}}$ are the concentration of the neutral and protonated species in the water phase.

For the SAMPL5 challenge, the objective was to determine $\log D$ for a set of 53 small molecules, by using state-of the art computational approaches. The experimental measurements were carried out at Genentech, according to a protocol previously described by Lin and Pease [11, 12]. The choice of organic solvent in the present experimental series was cyclohexane. Since distribution coefficients are implicitly related to solvation free energies, such a challenge also provides an insight into solvation free energy estimations and therefore loans itself to be addressed using molecular mechanics trajectory based alchemical free energy methods. This was the method of choice in this paper with computations carried out using the *Sire/OpenMM 6.3 (SOMD)* [13, 14] software. *SOMD* is a simulation tool that allows to run alchemical free energy calculations on GPUs, where *OpenMM* serves as the MD engine and *Sire* provides a set of molecular libraries on top of that. The choice of using trajectory based alchemical methods was partially motivated by the previously reported success with simple molecules such as caffeine (**80**) that were treated with general molecular mechanics force fields [15]. The motivation was also to understand at which point these methods currently fail when faced with larger and more chemically complex molecules such as rifampicin (**83**) or reserpine (**65**). The SI includes all structures corresponding to the numbered molecules discussed in the manuscript.

## Theory and methods

### Computing distribution coefficients: model A, B, C, and D

**Fig. 1:**
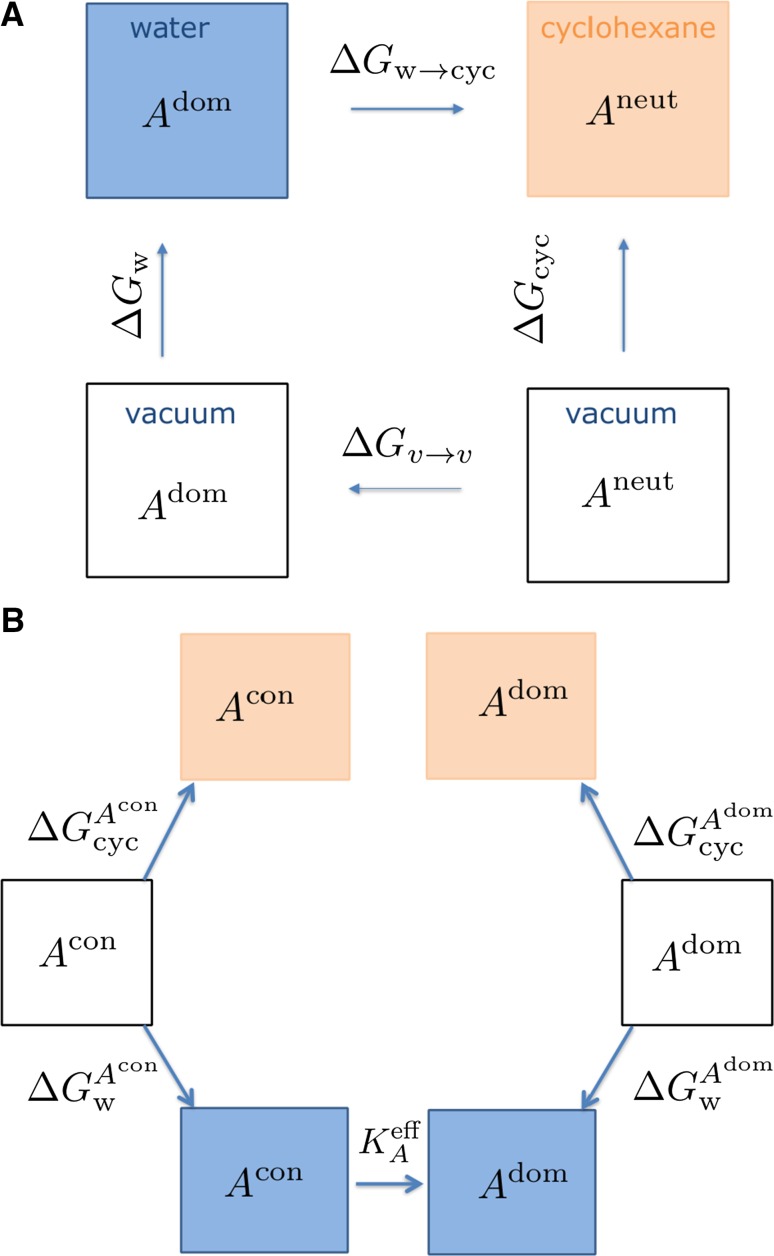
**A** Diagram of the *dominant species approximation*, **B** Diagram of the two-species approximation. Symbols are defined in the main text

The distribution coefficient $\log D$ is given by Eq. 2. Working with ionizable species gives rise to the complication that multiple protonation states need to be considered. To simplify protocols the approximation was made that a given molecule is predominantly in a single state (that may or may not be charged) in the water phase and in a neutral charge state in the organic phase. This approximation will be referred to as the *dominant species approximation*. A schematic diagram of the *dominant species approximation* can be found in Fig. 1a. This means a change in Gibbs free energy of a molecule A between a water phase and an organic phase (here cyclohexane), neglecting changes in activity coefficients, is given by:

3 $$\Delta G_{\mathrm{w \rightarrow cyc}} = -\beta ^{-1}\ln \frac{[A]_{\mathrm{cyc}}^{\mathrm{neut}}}{[A]^{\mathrm{dom}}_{\mathrm{w}}},$$

where $\beta$ is the inverse temperature given by $\beta = 1/k_BT$, $[A]_{\mathrm{cyc}}^{\mathrm{neut}}$ is the concentration of a neutral species in cyclohexane, and $[A]^{\mathrm{dom}}_{\mathrm{w}}$ is the concentration of the dominant species in water at pH 7.4. This leads to a definition of $\log D$, that depends on the free energy change between the organic phase and water phase of molecule A.

4 $$-\frac{1}{2.303}\beta \Delta G_{w\rightarrow \mathrm{cyc}} = \log \frac{[A]_{\mathrm{cyc}}^{\mathrm{neut}}}{[A]^{\mathrm{dom}}_{\mathrm{w}}} = \log D.$$

**Fig. 2:**
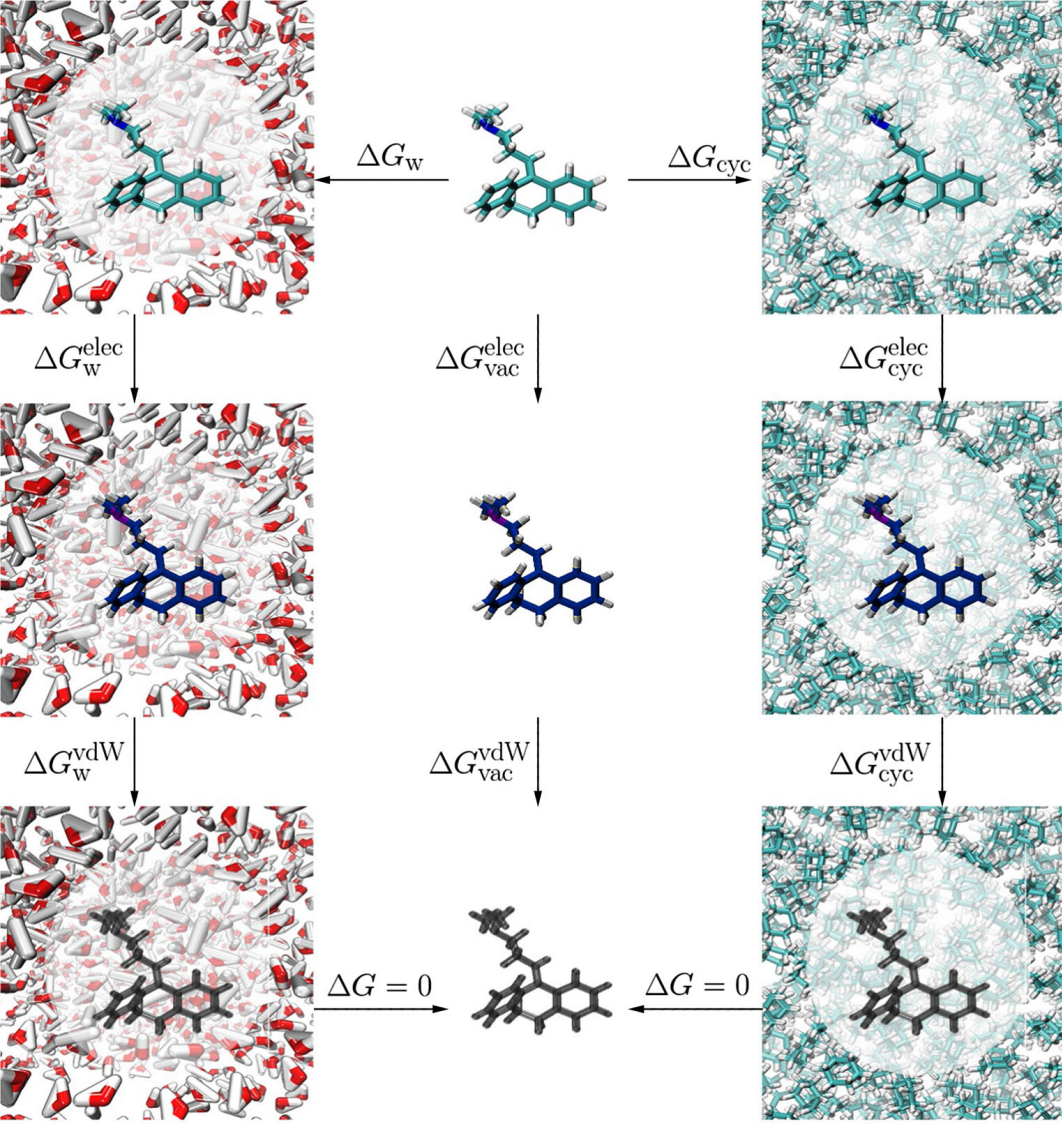
Thermodynamic cycle for $\log D$ calculation. First the atoms’ partial charges are turned off retrieving $\Delta G^{\mathrm{elec}}_{\mathrm{w}}$, $\Delta G^{\mathrm{elec}}_{\mathrm{vac}}$ and $\Delta G^{\mathrm{elec}}_{\mathrm{cyc}}$ in water, vacuum and cyclohexane phase respectively. This step is referred to as the ’discharging step’ in the main text. Then, van der Waals terms are switched off and $\Delta G^{\mathrm{vdW}}_{\mathrm{w}}$, $\Delta G^{\mathrm{vdW}}_{\mathrm{vac}}$ and $\Delta G^{\mathrm{vdW}}_{\mathrm{cyc}}$ are calculated in each phase. This step is referred to as the ’vanishing step’ in the main text. The diagram assumes no change in protonation state between solvated and vacuum phases

The next task is to compute $\Delta G_{w\rightarrow \mathrm{cyc}}$ from a series of simulations. The basic idea is summarized in Fig. 2 with a series of thermodynamic cycles. The goal is to compute the free energy of solvation in water and cyclohexane, such that:

5 $$\Delta G_{\mathrm{w}\rightarrow {cyc}}=\Delta G_{\mathrm{cyc}}-\Delta G_{\mathrm{w}} -\Delta G_{\mathrm{v\rightarrow v}}.$$

Each of the individual solvation free energies are computed using an annihilation method performed twice as shown in Fig. 2 and given by:

6 $$\Delta G_{\mathrm{solv}}^{\mathrm{model\, A}} = \Delta G_{\mathrm{solv}}^{\mathrm{elec}}+\Delta G_{\mathrm{solv}}^{\mathrm{vdW}}-(\Delta G_{\mathrm{vac}}^{\mathrm{elec}}+\Delta G_{\mathrm{vac}}^{\mathrm{vdW}})+\Delta G_{\mathrm{FUNC}},$$

where the identifier *solv* is either cyclohexane or water. The different free energy terms correspond to the discharging step, i.e. $\Delta G_{\mathrm{solv}}^{\mathrm{elec}}$ and $\Delta G_{\mathrm{vac}}^{\mathrm{elec}}$ in either solvent and vacuum respectively and the vanishing step in which the Lennard Jones terms are turned off in the annihilation protocol. The vanishing free energies in solvent and vacuum are given by $\Delta G_{\mathrm{solv}}^{\mathrm{vdW}}$ and $\Delta G_{\mathrm{vac}}^{\mathrm{vdW}}$, respectively. The correction term $\Delta G_{\mathrm{FUNC}}$ is used to account for using Barker-Watts reaction field (BWRF) electrostatics in the water and cyclohexane phase (see below). The term $\Delta G_{\mathrm{v \rightarrow v}}$ is the free energy change for converting molecule $A^\mathrm{neut}$ into $A^\mathrm{dom}$ in vacuum. This term is null if neut and dom are the same species. This term was also neglected for the cases where neut and dom species differ for the SAMPL submissions and the consequences are discussed in the results section.

In the actual simulations an alchemical approach is used to achieve the discharging and vanishing step (Fig. 2) [16]. To this end, an artificial parameter, $\lambda$, is introduced that modifies the potential of the molecule linearly to account for the decoupling. $\lambda$ is defined over the interval [0,...,1], creating intermediate states, referred to as alchemical states, between each transformation. Using the multistate Bennet’s acceptance ratio (MBAR) [17], a free energy difference between $\lambda =0$ and $\lambda =1$ can be used to evaluate the appropriate terms of Eq. 6.

In both solvated phases, the system’s Coulombic interactions are calculated based on BWRF. Thus, two different dielectric constant are adopted for water and cyclohexane simulations. However, for simulations in vacuum a reaction-field is inappropriate and instead a Coulombic potential without cutoffs was employed. Because a reaction-field is applied to all intra and intermolecular pairwise interactions, this leads to an inconsistent description of the intramolecular electrostatic interactions of the solute in the solvated and vacuum simulations.

Therefore to enable meaningful comparison of computed free energy changes, a free energy correction term $\Delta G_{\mathrm{FUNC}}$ was evaluated to treat intramolecular Coulombic interactions consistently between solvated and vacuum legs of the thermodynamic cycle depicted in Fig. 2. The $\Delta G_{\mathrm{FUNC}}$ term is obtained via post-processing the $\lambda =0.0$ trajectories of the discharging step of a solvated simulation and use of the Zwanzig relation [18]:

7 $$\Delta G_{\mathrm{FUNC}} = -\beta ^{-1}\ln \langle \exp [-\beta (U_{\mathrm{ic,nc}}({\mathbf {r}}) - U_{\mathrm{ic,sim}}({\mathbf {r}}))]\rangle _{\mathrm{sim}},$$

where $U_{\mathrm{ic,nc}}({\mathbf {r}})$ is the solute intramolecular electrostatic potential that depends on the coordinates ${\mathbf {r}}$ of the solute and Coulomb’s law. $U_{\mathrm{ic,sim}}({\mathbf {r}})$ is the intramolecular electrostatic potential term as computed during the simulation with a BWRF cutoff. Evaluation of the free energies according to Eq. 6 and then using these to evaluate $\log D$ according to Eq. 4 will be referred to as *model A.*

*Model B* is given by:

8 $$\Delta G_{\mathrm{solv}}^{\mathrm{model\,B}} =\Delta G_{\mathrm{solv}}^{\mathrm{model\, A}} +\Delta G^{\mathrm{cyc}}_{\mathrm{LJLRC}}-\Delta G^{\mathrm{w}}_{\mathrm{LJLRC}}.$$

Equation 8 is an extension to *model A* that takes a long range dispersion corrections $\Delta G^{\mathrm{solv}}_{\mathrm{LJRC}}$, derived by Shirts et al. [19], into account. This dispersion correction can readily be computed from a simulated trajectory using the Zwanzig relation:

9 $$\Delta G^{\mathrm{solv}}_{\mathrm{LJLRC}}=-\beta ^{-1}\ln \langle \exp [-\beta (U_{\mathrm{LJ,long}}({\mathbf {r}})-U_{\mathrm{LJ,sim}}({\mathbf {r}}))]\rangle _{\mathrm{solv}} + U_{\mathrm{LJ,ana}},$$

where $U_{\mathrm{LJ,long}}$ is the Lennard Jones energy calculated by increasing the long range cutoff and $U_{\mathrm{LJ,ana}}$ is an analytical correction for extending the long range cutoff to infinity. By post-processing each end state trajectory in the vanishing step of either solvated phase, the Lennard Jones potential, $U_{\mathrm{LJ,long}}$, is recalculated for each snapshot of all the solute and solvent molecule with an increased cutoff radius that is set to $r_{c,\mathrm{long}}=0.95 \min (L_x,L_y,L_z)/2$ where $L_x$, $L_y$, $L_z$ are the box edges length at the beginning of the simulation. The scaling factor accounts for small fluctuations in box size that could have produced reduced box edges in the generated trajectory. The additional contribution of the long range potential $U_{\mathrm{LJ,ana}}$ to Eq. 9 is evaluated as follow:

10 $$U_{\mathrm{LJ,ana}} = 8\pi \rho \sum _{\mathrm{i}}^{N_{\mathrm{sol}}}\sum _{\mathrm{j}}^{N_{\mathrm{solv}}}\left[ \frac{\epsilon _{\mathrm{ij}}\sigma _{\mathrm{ij}}^{12}}{9r_{\mathrm{c,long}}^9} - \frac{\epsilon _{\mathrm{ij}}\sigma _{\mathrm{ij}}^6}{3r_{\mathrm{c,long}}^3}\right] ,$$

where $\rho$ is the solvent density in mol$\cdot {\AA} ^{-3}$, $N_{\mathrm{sol}}$ is the total number of atoms of the solute molecule, $N_{\mathrm{solv}}$ the number of solvent molecules, $\epsilon _{\mathrm{ij}}$ is the Lennard Jones well depth, expressed in $\hbox {kcal} \cdot \hbox {mol}^{-1}$, and $\sigma _{\mathrm{ij}}$ is the Lennard Jones distance, in Å, calculated with the Lorentz-Berthelot combining rule [20]. The Lennard Jones parameters for both the cyclohexane solvent and water are discussed elsewhere. Equation 10 is derived by assuming that the box size is infinitely large and that the radial distribution function $g({\mathbf {r}})=1$ for distances greater than ${r}_{\mathrm{c,long}}$.

*Model C* takes corrections for the discharging free energy step into consideration. This is based on the work by Reif and Oostenbrink [21], Rocklin et al. [22], and earlier work from Kastenholz and Hünenberger [23, 24]. Here corrections on the free energy estimation for a BWRF atom based cutoff for the discharging step were derived. Net charge free energy calculations are affected by several finite size artefacts [21, 22]. To be computationally efficient periodic boundary conditions along with an effective Coulombic potential are employed, which introduces artefacts that can be sizable for simulations of charged species [25, 26]. Additionally, solvent models typically do not exactly reproduce the experimental dielectric permittivity, i.e. for TIP3P water under the conditions simulated here the dielectric constant is 82 [27] as opposed to an experimental value of 78.3. To correct for these source of errors a correction term $\Delta G_{\mathrm{POL}}$ was calculated as:

11 $$\begin{aligned} \Delta G_{\mathrm{POL}} = \Delta G_{\mathrm{NP}}^{\mathrm{Coul}} - \Delta G_{\mathrm{RF}}^{\mathrm{Coul}}, \end{aligned}$$

where $\Delta G_{\mathrm{NP}}^{\mathrm{Coul}}$ is the electrostatic free energy due to Coulombic interactions under non-periodic conditions, as obtained by solving the Poisson equation with the software *APBS* [28]. $\Delta G_{\mathrm{RF}}^{\mathrm{Coul}}$ is the electrostatic free energy obtained solving the Poisson equation under BWRF and periodic boundary conditions, using a custom code kindly given to us by Hünenberger [29]. A second source of error occurs in the present molecular simulations due to the use of an atom-based cutoff to compute solute-solvent interactions. This summation scheme causes an apparent solvation of negatively charged species and a de-solvation of positively charged molecules [21, 23, 24]. For atom-based BWRF conditions a $\Delta G_{\mathrm{PSUM}}$ correction term was evaluated as:

12 $$\begin{aligned} \Delta G_{\mathrm{PSUM}} = &-\frac{N_A}{6\epsilon _0} Q_{\mathrm{mol}} \gamma _{\mathrm{s}} \left[ \left( \frac{2(\epsilon _{\mathrm{BW}} + 1 )}{2\epsilon _{\mathrm{BW}} + 1 } \times \frac{ \langle N_{\mathrm{S}}(r_{\mathrm{c}} ) \rangle }{ 4\pi \frac{r_{\mathrm{c}}^3}{3} } \right)\right.\\ &+ \left.\frac{3}{2\epsilon _{\mathrm{BW}} + 1 } \right] , \end{aligned}$$

where $N_{\mathrm{A}}$ is Avogadro number, $\epsilon _0$ is the experimental permittivity for the solvent, $\epsilon _{\mathrm{BW}}$ is the dielectric constant of the water model used, $\gamma _{\mathrm{s}} = \sum _{\mathrm{i=1}}^{\mathrm{N}} q_{\mathrm{i}}{\mathbf {r}}_{\mathrm{i}}$ is the trace of the quadrupole-moment tensor of the solvent model, where the sum is over all *N* atoms in a solvent molecule, $q_{\mathrm{i}}$ is the charge of the *i*-th atom in a solvent molecule, ${\mathbf {r}}_{\mathrm{i}}$ is the coordinate vector, $Q_{\mathrm{mol}}$ is the net charge of the solute, $r_{\mathrm{c}}$ is the reaction field cutoff length and $\langle N_{\mathrm{s}}(r_{\mathrm{c}}) \rangle$ is the average number of solvent molecules within $r_{\mathrm{c}}$. This leads to a free energy evaluation of *model C* according to:

13 $$\begin{aligned} \Delta G_{\mathrm{solv}}^{\mathrm{model\,C}}=\Delta G_{\mathrm{solv}}^{\mathrm{model\,B}}+\Delta G_{\mathrm{POL}}+\Delta G_{\mathrm{PSUM}} . \end{aligned}$$

*Model D* is the same as *model C*, but applying the correction introduced for *model C* only to charged species.

### Two-species assumption

After the results of the competition were revealed an attempt was made to improve on the estimations obtained by introducing an alternative to the *dominant species approximation*. Generally, assuming all activity coefficients to be unity, the distribution coefficient $\log D$ is given by:

14 $$\begin{aligned} \log D = \log \left( \frac{ \sum _{\mathrm{i}}^{\mathrm{N_q}} \sum _{\mathrm{j}}^{\mathrm{N_{taut}}} [A_{\mathrm{j}}]^{\mathrm{i}}_{\mathrm{cyc}} }{ \sum _{\mathrm{k}}^{\mathrm{N_q}} \sum _{\mathrm{l}}^{\mathrm{N_{taut}}} [A_{\mathrm{l}}]^{\mathrm{k}}_{\mathrm{w}} } \right) , \end{aligned}$$

where the sums are extends over all the possible protonation $(N_q)$ and tautomeric state $(N_{\mathrm{taut}})$ *i* and *j* in cyclohexane and *k* and *l* in water phase, for a molecule *A*. Then, the concentration of the most populated species in water at pH 7.4 is given by:

15 $$\begin{aligned}{}[A]^{\mathrm{dom}}_{\mathrm{w}}= f^{\mathrm{chemicalize}}(A^{\mathrm{dom}} _{\mathrm{w}}) \times [A]_{\mathrm{tot}}, \end{aligned}$$

where $f^{\mathrm{chemicalize}}(A^{\mathrm{dom}} _{\mathrm{w}})$ is the fraction of the dominant species $A^{\mathrm{dom}} _{\mathrm{w}}$ predicted by the software ChemAxon [30] at pH 7.4. $[A]_{\mathrm{tot}}$ is by convention set to 1M. Note that the fraction of dominant species is determined by considering potentially multiple equilibria between different charged states and tautomers.

We now assume that the only other species in solution is the conjugate pair of $A^{\mathrm{dom}} _{\mathrm{w}}$, which will be denoted $A^{\mathrm{con}} _{\mathrm{w}}$. If there are multiple ionisable sites $A^{\mathrm{con}} _{\mathrm{w}}$ is taken to be the conjugate pair that is expected to have the highest population on the basis of the pKa values of each ionisable site. Thus:

16 $$\begin{aligned}{}[A]^{\mathrm{con}}_{\mathrm{w}} = 1 - f^{\mathrm{chemicalize}}(A^{\mathrm{dom}} _{\mathrm{w}}) \times [A]_{\mathrm{tot}}. \end{aligned}$$

Since only two species are considered, Eq. 14 reduces to:

17 $$\begin{aligned} \log D=\log \left( \frac{[A]^{\mathrm{con}}_{\mathrm{cyc}} + [A]^{\mathrm{dom}}_{\mathrm{cyc}} }{ [A]^{\mathrm{con}}_{\mathrm{w}} + [A]^{\mathrm{dom}}_{\mathrm{w}} } \right) . \end{aligned}$$

And since pH, $[A]^{\mathrm{con}}_{\mathrm{w}}$ and $[A]^{\mathrm{dom}}_{\mathrm{w}}$ are known, an effective $\hbox {pKa}^{\mathrm{eff}}$ can be defined:

18 $$\begin{aligned} \mathrm{pKa}^{\mathrm{eff}} = \mathrm{pH} - \log \frac{[A]^{\mathrm{dom}}_{\mathrm{w}}}{[A]^{\mathrm{con}}_{\mathrm{w}}}. \end{aligned}$$

where for simplicity in the notation it has been assumed that the dominant form is the base and the conjugate form the acid. Although $A^{\mathrm{con}}_{\mathrm{w}}$ and $A^{\mathrm{dom}}_{\mathrm{w}}$ are conjugate pairs, the term effective pKa is used because the relative concentrations of the two species are set by $f^{\mathrm{chemicalize}}(A^{\mathrm{dom}} _{\mathrm{w}})$, a quantity that was derived by considering co-existence of more than two species.

Rearrangement of Eq. 18 and insertion in Eq. 17 leads to:

19 $$\log D =\log \left( P_{\mathrm{A^{\mathrm{con}}}} \left( 1+ \frac{10^{-pKa^{\mathrm{eff}}}}{10^{-{\mathrm{pH}}}} \right) ^{-1} + P_{\mathrm{A^{\mathrm{dom}}}} \left( 1+\frac{10^{-{\mathrm{pH}}} }{10^{-\mathrm{pKa^{\mathrm{eff}}}}} \right) ^{-1} \right) ,$$

where $P_{\rm A^{\rm con}}=\frac{[A]^{\rm con}_{\rm cyc}}{[A]^{\rm con}_{\rm w}}$ and $P_{\rm A^{\rm dom}}=\frac{[A]^{\rm dom}_{\rm cyc}}{[A]^{\rm dom}_{\rm w}}$. Equation 19 may be solved by computing P values for $A^{\mathrm{con}}$ and $A^{\mathrm{dom}}$ from calculated transfer free energies for each species, and knowledge of effective pKa and pH values. This approach will be referred to as the *two-species assumption* since it enables the consideration of up to two chemical states of a molecule in each phase.

For molecules that contain a single ionisable site and have no alternative tautomeric forms $pKa^{\mathrm{eff}}=pKa$, and if additionally ${P_{\mathrm{A^{\mathrm{con}}}}}\gg{P_{\mathrm{A^{\mathrm{dom}}}}}$ then Eq. 19 simplifies to the more commonly used approximation [31]:

20 $$\log D = \log \left[ P_{\mathrm{A^{\mathrm{con}}}} \left( 1 + \frac{10^{\mathrm{-pKa}}}{10^{\mathrm{-pH}}} \right) ^{-1} \right].$$

### Datasets

The Minnesota Solvation Database [32] is a collection of 3037 experimental solvation and transfer free energies. Therefore, it constitutes a useful resource to study new methods for free energy calculations. In the present study 14 small molecules were selected from this database, shown in Fig. 1 of the supplementary information (SI), chosen based on similar moieties present in the SAMPL5 dataset. This data set was then used to asses accuracy of solvation free energy calculations use Sire/OpenMM [13, 14], with the different proposed methods for the SAMPL5 study and therefore served as an initial test dataset. This was of interest since solvation free energies are used to eventually compute $\log D$. No distribution coefficient data between cyclohexane and water was available for the chosen molecules from Minnesota Solvation Database [32] and therefore it was difficult to assess the accuracy of the *dominant species approximation* for the $\log D$ calcualtions prior to submission. The SAMPL5 dataset consists of 53 drug-like molecules, depicted in Fig. 2 of the SI, and was provided by the organisers as mol2 or sdf files. Experimental facilities for the distribution coefficient dataset were generously provided by Genentech, and measurements were done according to the protocol described by Lin and Pease [11, 12].

### Simulation setup

All molecules were parametrized with the general Amber force field (GAFF) [33], solvated in both cubic boxes of TIP3P water molecules [34] and GAFF cyclohexane. Each system was initially energy minimized for 100 cycles by using the steepest descent method with harmonic positional restraints using a force constant of 10 $\hbox {kcal}\cdot \hbox {mol}^{-1}$ Å$^{-2}$ applied to the whole water molecules or cyclohexane molecules respectively, allowing the solute to relax. Secondly, an NVT equilibration of 200 ps at 298 K, following an NPT equilibration at 1 atm with Amber module Sander [35] were carried out. Finally, a 2 ns simulation in the NPT ensemble was run with Sire/OpenMM 6.3 (rev 15.1) [13, 14], to reach a final density of 1 g/cc and 0.7 g/cc for water and cyclohexane respectively. Then, coordinate files were retrieved with CPPTRAJ [36]. This was the protocol used for all uncharged species in the *dominant species approximation*. From the mol2 file the topology and the coordinates for vacuum simulations were created with the help of tleap. For each molecule only the most populated state was considered, based on pKa calculation with ChemAxon [30] at pH 7.4 for the *dominant species approximation*. Where necessary, molecules were protonated with Maestro (v.10.1.012, rel 2015-1, Schrödinger) [37]. Then, Antechamber 14 [35] was run to obtain AM1-BCC charges [38]. In the case of charged species the molecules were then re-solvated and underwent the same procedure as described above for the uncharged species.

In the case of the test dataset, consisting of the 14 chosen molecules of the Minnesota Solvation Database [32], all initial structures were sketched with Maestro, parametrized with the general Amber force field [33], and solvated in rectangular boxes of TIP3P water molecules and GAFF cyclohexane, with a minimum distance between the solute and the box edges of 12 Å.

### Alchemical free energy production simulations

Each discharging step was divided into nine equidistant $\lambda$ windows. For the vanishing step, 11 equidistant $\lambda$ windows were used, and an additional window was added at 0.950, to capture large fluctuations in the free energy changes towards the end of the decoupling process. Each $\lambda$ window was run for 2 ns in the organic and aqueous phase, except molecules **7**, **13**, **19**, **24**, **42**, **56**, **65**, **71**, **88**, and **92**, whose vanishing step was run for 6 ns, to improve the precision of the computed free energy changes. Additionally, for vacuum simulation each $\lambda$ window was run for 0.8 ns. A velocity-Verlet integrator was employed with a time step of 4 fs using a hydrogen mass repartitioning scheme (HMR) [39] by constraining all bonds. All simulations were performed at 298 K and 1 atm in an NPT ensemble, using an atom-based Barker Watts reaction field [40] with a dielectric constant of 82 for the water phase and a dielectric constant of 1.0 for the cyclohexane phase. The non-bonded interactions cutoff was set to 12 Å and periodic boundary conditions were imposed. An Andersen thermostat with a coupling constant of $10\,\hbox {ps}^{-1}$ [41] assured the temperature control, while a Monte Carlo barostat was used for pressure control, attempting isotropic box edge scaling every 25 time steps.

*Estimation of* $\log D$ *for models A, B, C and D*

All solvation free energy estimates for the Minnesota test data set were done using MBAR [17]. The estimates are based on a single simulation and errors are obtained from the asymptotic variance estimator as implemented in pymbar [42], where uncorrelated samples were drawn from the generated trajectories using the timescale module in pymbar. Errors were then propagated using standard rules of error propagation. Propagated errors are reported as error bars in the results section only for the Minnesota database data.

All free energy estimates for the SAMPL5 dataset from both the discharging and vanishing step needed for the computation of $\log D$ for any of the methods were done using MBAR [17]. A different methodology was used to estimate errors for this dataset. Here all solvation free energies in both water and cyclohexane were computed twice using different initial assignments of velocities drawn from the Maxwell-Boltzmann distribution. Computed distribution coefficients are reported as the average of the two independent simulations for which $\log D$ was calculated, and statistical uncertainties were calculated according:

21 $$\begin{aligned} \mathrm{err}(\Delta G) = \frac{\sigma }{\sqrt{n}}, \end{aligned}$$

where $\sigma$ is the standard deviation of both runs and $n=2$, unless otherwise stated. These are the error bars reported in the results section for all of the SAMPL5 challenge data.

The computed distribution coefficients according to each model are then correlated to experimental values using the determination coefficient $R^2$ and the accuracy of the computed value itself is measured using the mean unsigned error (MUE). To gain insight into the distribution of the two different measures a bootstrapping scheme is used in which each point is considered to be a normal distribution with its mean given by the computed free energy and $\sigma$ the associated computed error. Ten thousand samples are then drawn from the artificial normal distributions for each data point and correlated with the experimental values, giving rise to a distribution of $R^2$ and MUE. The resulting distributions are typically not symmetric around the mean and uncertainties in the dataset metrics are reported with a 95$\%$ confidence interval written in the follow way $z-<\mu <z+$, where $z-$ is the lower bound and $z+$ the upper bound of the confidence interval and $\mu$ the mean of the distribution. All simulation input files and post processing scripts needed for reproducing the results as well as results files can be found in a github repository https://github.com/michellab/Sire-SAMPL5.

## Results

### Solvation free energies of the Minnesota dataset

**Fig. 3:**
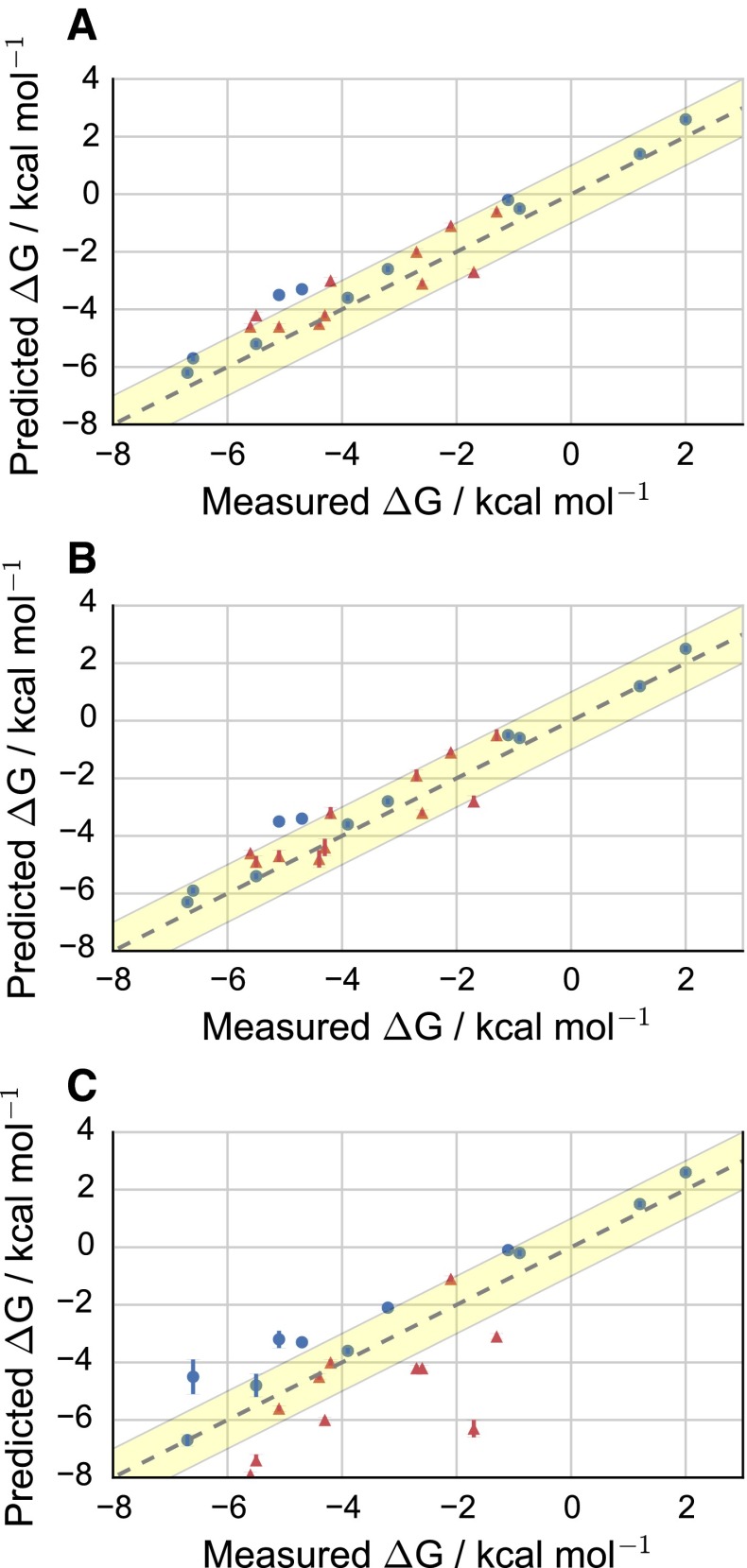
Computed solvation free energy in water (*blue circles*) and in cyclohexane (*red triangles*) for neutral compounds selected from the Minnesota Solvation Database [32] according to *models A* (**A**), *B* (**B**), and *C* (**C**). *Model D* is not shown since only neutral species are plotted, meaning that *model D* is equivalent to *model B*. The *grey dashed line* assumes a perfect correlation and the *yellow shaded* interval corresponds to an error of 1 $\hbox {kcal}\cdot\hbox {mol}^{-1}$

Figure 3 shows a scatter plot of the solvation free energies in water $\Delta \hbox {G}_{\mathrm{w}}$ and cyclohexane $\Delta \hbox {G}_{\mathrm{cyc}}$ for all neutral molecules of the dataset chosen from the Minnesota solvation database [32], reported in Table 1.

Both *models A* and *B* yield similar results for neutral molecules in water, with $\hbox {R}^2 = 0.96<0.97<0.98$ and $\hbox {MUE}=0.65<0.71<0.77\,\hbox {kcal}\cdot \hbox {mol}^{-1}$ and $0.52<0.57<0.64\,\hbox {kcal}\cdot \hbox {mol}^{-1}$ respectively, as shown in Table 1, and in panel A and B of Fig. 3 respectively. Inclusion of the two charged molecules trimethylammonium and acetate causes larger deviations from the experimental data as clearly seen when considering the whole dataset of Table 1, giving rise to a $\hbox {MUE}=3.58<3.63<3.69\,\hbox {kcal}\cdot \hbox {mol}^{-1}$ for *model A*, while a tiny improvement is introduced for *model B* $(\hbox {MUE}=3.45<3.51<3.57\,\hbox {kcal}\cdot \hbox {mol}^{-1})$. The results have worsened mainly because of the very large discrepancy between the computed and measured hydration free energy of trimethylammonium (−24.7 vs −61.4 $\hbox {kcal}\cdot \hbox {mol}^{-1}$).

The addition of charging corrections (*model C*) gives better agreement with experimental data for the whole dataset, with a $\hbox {MUE}=0.95<1.07<1.19\,\hbox {kcal}\cdot \hbox {mol}^{-1}$ and $\hbox {R}^2 = 0.98<0.99<1.00$ and *model D* results in the best prediction ($\hbox {MUE}=0.71<0.77<0.84\,\hbox {kcal}\cdot \hbox {mol}^{-1}$ and $\hbox {R}^2 = 0.98< 0.99< 1.00$). Figure 3c shows the results of adding the charging corrections of *model C* to all neutral molecules. *Model D* is only depicted as the subdataset of the neutral molecules in Fig. 3, and is the equivalent of panel B.

**Table 1:** Computed solvation free energy for Minnesota dataset [32]. $\Delta G_{\mathrm{w}}$ is the absolute free energy of hydration and $\Delta G_{\mathrm{cyc}}$ the absolute free energy of solvation in cyclohexane, both expressed in $\hbox {kcal}\cdot \hbox {mol}^{-1}$.* A, B, C* and *D* refer to the *model* described in section 2. MUE and $\hbox {R}^2$ denotes the mean unsigned error $(\hbox {kcal}\cdot \hbox {mol}^{-1})$ and the determination coefficient for the whole dataset. MUE neutral and $\hbox {R}^2$ shows the mean unsigned error $(\hbox {kcal}\cdot \hbox {mol}^{-1})$ and determination coefficient for the neutral species only. *Model D* for solvation free energies in cyclohexane is the same as *model B*. The notation z- $<\mu<$ z+ signifies 95 % confidence intervals computed from the bootstrapping of the data

Molecule	$\Delta G_{\mathrm{w}}$	A	B	C	D
Cyclohexane	1.2	1.4 ± 0.1	1.2 ± 0.1	1.5 ± 0.1	1.2 ± 0.1
Benzene	−0.9	−0.5 ± 0.1	−0.6 ± 0.1	−0.2 ± 0.1	−0.6 ± 0.1
Acetic acid	−6.7	−6.2 ± 0.1	−6.3 ± 0.1	−6.7 ± 0.2	−6.3 ± 0.1
Trimethylamine	−3.2	−2.6 ± 0.1	−2.8 ± 0.1	−2.1 ± 0.1	−2.8 ± 0.1
Chlorobenzene	−1.1	−0.2 ± 0.1	−0.5 ± 0.1	−0.1 ± 0.1	−0.5 ± 0.1
Methanol	−5.1	−3.5 ± 0.1	−3.5 ± 0.1	−3.2 ± 0.3	−3.5 ± 0.1
n-Propane	2.0	2.6 ± 0.1	2.5 ± 0.1	2.6 ± 0.1	2.5 ± 0.1
Pyridine	−4.7	−3.3 ± 0.1	−3.4 ± 0.1	−3.3 ± 0.1	−3.4 ± 0.1
Phenol	−6.6	−5.7 ± 0.1	−5.9 ± 0.1	−4.5 ± 0.6	−5.9 ± 0.1
Acetone	−3.9	−3.6 ± 0.1	−3.6 ± 0.1	−3.6 ± 0.1	−3.6 ± 0.1
Aniline	−5.5	−5.2 ± 0.1	−5.4 ± 0.1	−4.8 ± 0.4	−5.4 ± 0.1
Trimethylammonium	−61.4	−24.7 ± 0.1	−24.8 ± 0.1	−61.4 ± 0.3	−61.4 ± 0.3
Acetate	−77.6	−74.8 ± 0.1	−74.9 ± 0.2	−81.1 ± 0.3	−81.1 ± 0.3
MUE		3.58 < 3.63 < 3.69	$3.45\,<\,3.51\,<\,3.57$	$0.95\,<\,1.07\,<\,1.19$	$0.71\,<\,0.77\,<\,0.84$
$\hbox {R}^2$		$0.85\,<\,0.86<0.87$	$0.85\,<\,0.86\,<\,0.87$	$0.98\,<\,0.99\,<\,1.00$	$0.98\,<\,0.99\,<\,1.00$
MUE neutral		$0.65<0.71<0.77$	$0.52\,<\,0.57\,<\,0.64$	$0.80\,<\,0.93\,<\,1.05$	$0.52\,<\,0.57\,<\,0.64$
$\hbox {R}^2$ neutral		$0.96\,<\,0.97\,<\,0.98$	$0.96\,<\,0.97\,<\,0.98$	$0.90\,<\,0.94\,<\,0.96$	$0.96\,<\,0.97\,<\,0.98$

Molecule	$\Delta \hbox {G}_{\mathrm{cyc}}$	A	B	C	
Cyclohexane	−4.4	−4.5 ± 0.1	−4.8 ± 0.3	−4.5 ± 0.1	
Benzene	−4.2	−3 ± 0.1	−3.2 ± 0.2	−4 ± 0.1	
Acetic acid	−1.7	−2.7 ± 0.1	−2.8 ± 0.2	−6.3 ± 0.3	
Trimethylamine	−2.6	−3.1 ± 0.1	−3.2 ± 0.1	−4.2 ± 0.1	
Chlorobenzene	−5.1	−4.6 ± 0.1	−4.7 ± 0.2	−5.6 ± 0.1	
Methanol	−1.3	−0.6 ± 0.1	−0.5 ± 0.2	−3.1 ± 0.1	
n-Propane	−2.1	−1.1 ± 0.1	−1.1 ± 0.1	−1.1 ± 0.1	
Pyridine	−4.3	−4.2 ± 0.1	−4.4 ± 0.3	−6 ± 0.1	
Phenol	−5.6	−4.6 ± 0.1	−4.6 ± 0.1	−7.9 ± 0.1	
Acetone	−2.7	−2 ± 0.1	−1.9 ± 0.2	−4.2 ± 0.1	
Aniline	−5.5	−4.2 ± 0.1	−4.9 ± 0.2	−7.4 ± 0.2	
MUE		$0.68\,<\,0.74\,<\,0.80$	$0.68\,<\,0.76\,<\,0.85$	$1.50\,<\,1.57\,<\,1.65$	
$\hbox {R}^2$		$0.74\,<\,0.77\,<\,0.81$	$0.69\,<\,0.74\,<\,0.79$	$0.37\,<\,0.43\,<\,0.49$	

Looking at the cyclohexane solvation free energies of *model A* and *model B* a similar trend with $\hbox {MUE}=0.68<0.74<0.80\,\hbox {kcal}\cdot \hbox {mol}^{-1}$ and $\hbox {MUE}=0.68<0.76<0.85$ respectively, along with an $\hbox {R}^2 = 0.74<0.77<0.81$ and $\hbox {R}^2 = 0.69<0.74<0.79$ can be observed, shown in panel A and B of Fig. 3 using red triangles. In contrast, *model C* shows a higher mean unsigned error $(\hbox {MUE} =1.50<1.57<1.65\,\hbox {kcal}\cdot \hbox {mol}^{-1})$ along with a lower determination coefficient $(\hbox {R}^2 = 0.37<0.43<0.49)$, see Fig. 3 **c**. In this case charging corrections fail to improve the estimations. As pointed out by Beauchamp et al. [43], the solvation of polar solutes in a non-polar solvent such as cyclohexane is expected to be systematically underestimated since the lack of polarisability yields a cyclohexane model with a static dielectric constant of cyclohexane of about 1.0, whereas experimental data indicates a value closer to 2.0. This is expected to cause a significant error in the computed solvation free energy of polar solutes in a non-polar solvent. In light of this argument, the present results are unexpected since the addition of correction terms that account for the experimental dielectric constant of cyclohexane yield results that are significantly worse (Table 1 *model C*) than the uncorrected results (Table 1 *model A*). Closer inspection of Table 1 confirms that solvation free energies of polar solutes in cyclohexane are slightly too positive for *model A*, but significantly too negative for *model C*.

### Dominant species model distribution coefficients

**Fig. 4:**
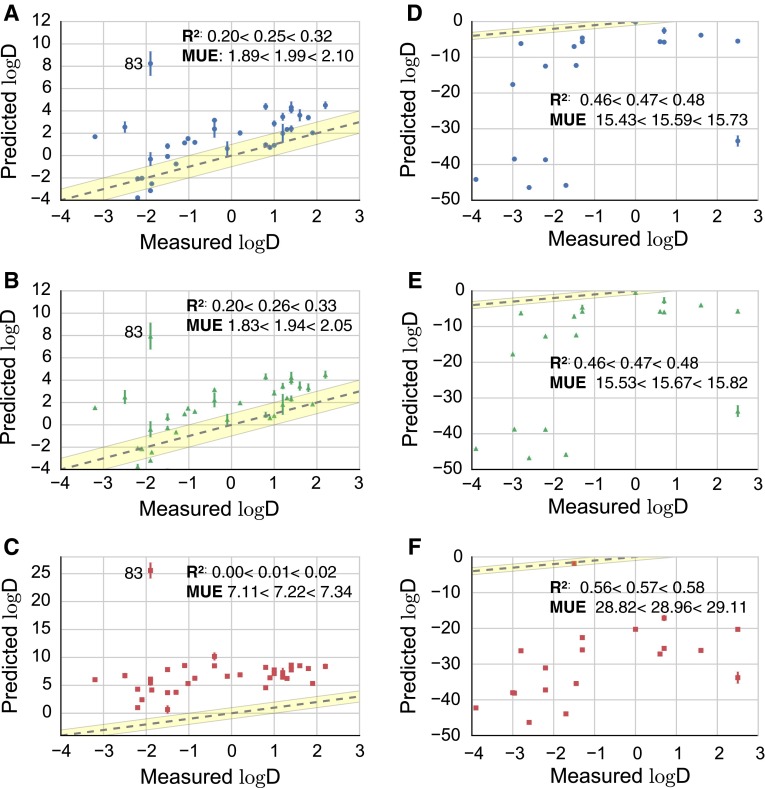
Scatter plots of computed $\log D$ for molecules modelled as neutral in water and in cyclohexane (**A**–**C**) and molecules modelled as charged in water and neutral in cyclohexane (**D**–**F**) molecules according to *model A* (*top*, *blue circles*), *model B* (*middle*, *green triangles*) and *model C* (*bottom*, *red squares*); MUE and $\hbox {R}^2$ values are given with 95 % confidence intervals and MUE in $\log D$ units. The *grey dashed line* assumes a perfect correlation and the *yellow shaded* interval corresponds to an error of 1 $\log D$

Next, *model A, B, C* and *D* were applied to all 53 molecules of the SAMPL5 challenge. Figure 4 compares $\log D$ predictions for each model for neutral and charged molecules. *Model D* is not shown, because it corresponds to *model B* for neutral species and *model C* for charged ones. Determination coefficient $\hbox {R}^2$ and MUE are summarized in Table 2. Solvation free energy results can be found in the SI.

Both *model A* and *B* yield similar results and are not statistically distinguishable from each other. This is illustrated with the bar and whiskers plot in Fig. 3 of the SI. Considering the whole dataset of molecules no differences arise between the two models with $\hbox {R}^2 = 0.26<0.27<0.28$ and $\hbox {MUE}=6.79<6.87<6.95\,\log D$ units for *model A* and $\hbox {MUE}=6.78<6.86<6.95\,\log D$ units for *model B* as Table 2 shows. The high MUE is mainly due to the ionizable species, where *model A* has a $\hbox {MUE}=15.45<15.59<15.74\,\log D$ units and *model B* $\hbox {MUE}=15.45<15.68<15.82\,\log D$ units. When only considering the set of neutral species, **83**, clearly visible in Fig. 4a and b, is the largest outlier, with a calculated $\log D=8.24\pm 1.09$, $7.94\pm 1.19$ for *model A* and *B* respectively, with respect to the experimentally measured $\log \hbox {D}=-1.9 \pm 0.4$. Such a discrepancy may be down to the large size and numbers of functional groups present in this molecule. Inspection of Fig. 4d and f makes it clear that predictions for charged species systematically and significantly deviate from experimental data. In particular, **60**, **10**, **11**, **26** and **15** are systematically wrongly predicted in all models, with $\log D$ values ranging between -40 and -50 and shown in the bottom left corner of Fig. 4 **d** and **f**.

**Table 2:** Comparison between $\hbox {R}^2$ and MUE for *model A, B, C* and *D* considering the whole dataset ($\hbox {R}^2$ and MUE) or neutral molecules ($\hbox {R}^2$ neutral and MUE neutral) or protonated species only ($\hbox {R}^2$ charged and MUE charged) for the *dominant species approximation*. All MUE are given in $\log D$ units. The notation z− $<\mu<$ z+ signifies 95 % confidence intervals taken from the bootstrapping of the data

	*Model A*	*Model B*
$\hbox {R}^2$	$0.26\,<\,0.27\,<\,0.28$	$0.26\,<\,0.27\,<\,0.28$
MUE	$6.79\,<\,6.87\,<\,6.95$	$6.78\,<\,6.86\,<\,6.95$
$\hbox {R}^2$ neutral	$0.20\,<\,0.25\,<\,0.32$	$0.20\,<\,0.27\,<\,0.34$
MUE neutral	$1.89 < 1.99 < 2.09$	$1.84\,<\,1.94\,<\,2.04$
$\hbox {R}^2$ charged	$0.46\,<\,0.47\,<\,0.48$	$0.46\,<\,0.47\,<\,0.48$
MUE charged	$15.45\,<\,15.59\,<\,15.74$	$15.54\,<\,15.68\,<\,15.82$

	*Model C*	*Model D*
$\hbox {R}^2$	$0.14\,<\,0.15\,<\,0.16$	$0.16\,<\,0.17\,<\,0.18$
MUE	$14.92\,<\,15.01\,<\,15.11$	$12.28\,<\,12.63\,<\,12.98$
$\hbox {R}^2$ neutral	$0.00\,<\,0.01\,<\,0.02$	$0.20\,<\,0.27\,<\,0.34$
MUE neutral	$7.11\,<\,7.22\,<\,7.94$	$1.84\,<\,1.94\,<\,2.04$
$\hbox {R}^2$ charged	$0.56\,<\,0.57\,<\,0.58$	$0.56\,<\,0.57\,<\,0.58$
MUE charged	$28.81\,<\,28.96\,<\,29.13$	$28.81\,<\,28.96\,<\,29.13$

The introduction of the charging corrections with *model C* do not statistically significantly improve the estimates, as shown in Fig. 3 of the SI, and the results obtained are not consistent with experimental values. A clear overestimation of the distribution coefficient is observed, with $\hbox {R}^2 = 0.14<0.15<0.16$ and $\hbox {MUE}=14.92<15.01<15.11\,\log D$ units for the entire dataset. In particular, both for neutral molecules and for charged molecules there is an increase in MUE with respect to *model A* and *model B* as shown in Fig. 4 **c** and **f** and Table 2. In Fig. 4 **c** the estimate for molecule **83** has clearly worsened after the application of charging corrections of *model C*, giving rise to virtually no correlation. Excluding molecule **83** gives a determination coefficient of neutral species with *model C* is $\hbox {R}^2 = 0.22<0.26<0.31$ and a MUE of $6.51<6.60<6.70\,\log D$ units. Again, GAFF seems to overly favor solvation of neutral molecules in hydrophobic media, and the addition of finite-size electrostatics corrections cause the solvation free energies to become even more negative. This generates a systematic bias in distribution coefficient predictions.

A slight improvement is reached with *model D*, whose $\hbox {R}^2 = 0.16<0.17<0.18$ and $\hbox {MUE} = 12.28<12.63<12.98\,\log D$ for the whole dataset, along with a statistically significant improvement with respect to *model C*. Overall, predictions with charging correction deviated significantly more from the experimental data, compared to *model A* and *B*.

Another source of error in the *dominant species approximation* is the neglect of the term $\Delta G_{\mathrm{v\rightarrow v}}$ present in Eq. 5 for molecules neutral and dominant species differ in cyclohexane and water. Attempts to evaluate this term were not made initially due to a lack of time to meet the submission deadline. However it is problematic to evaluate rigorously this term with alchemical methods and a classical potential energy function. Given these difficulties and the poor results obtained for charged molecules, further use of the *dominant species approximation* is not recommended.

### Two-species approximation

**Fig. 5:**
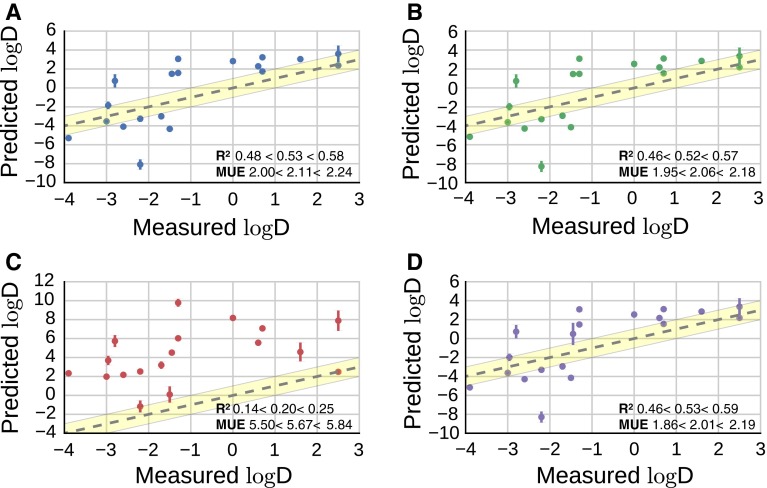
*Scatter plot* of $\log D$ estimation with the two-species model, for the subset of molecules predicted to co-exist in charged and neutral forms in aqueous phase, according to *model A* (*blue circles*), *model B* (*green circles*), *model C* (*red circles*), *model D* (*purple circles*)

Given the poor performance of the *dominant species approximation*, the *two-species approximation* was retrospectively applied to the whole batch of molecules. Fig. 5a and d shows the scatter plot of $\log D$ predictions for charged species only. A comparison between all models to understand whether one model is statistically significantly better than any other is given in Fig. 4 of the SI. Determination coefficient and MUE are shows in Table 3. Solvation free energy results for charged molecules are summarized in the SI. The $\log D$ predictions for non-ionizable compounds are identical to those obtained with the *dominant species approximation*.

**Table 3:** Comparison between $\hbox {R}^2$ and MUE for *model A, B, C* and *D* considering the whole dataset ($\hbox {R}^2$ and MUE) or protonated species only ($\hbox {R}^2$ charged and MUE charged) for the two-species approximation. All MUE give in $\log D$ units. The notation z − $<\mu<$ z+ signifies 95 % confidence intervals taken from the bootstrapping of the data

	*Model A*	*Model B*
$\hbox {R}^2$	$0.36\,<\,0.40\,<\,0.45$	$0.35\,<\,0.40\,<\,0.45$
MUE	$1.95\,<\,2.03\,<\,2.11$	$1.90\,<\,1.98\,<\,2.06$
$\hbox {R}^2$ charged	$0.48\,<\,0.53\,<\,0.58$	$0.46\,<\,0.52\,<\,0.57$
MUE charged	$2.00\,<\,2.11\,<\,2.24$	$1.95\,<\,2.06\,<\,2.18$

	*Model C*	*Model D*
$\hbox {R}^2$	$0.05\,<\,0.07\,<\,0.09$	$0.35\,<\,0.40\,<\,0.45$
MUE	$6.57\,<\,6.67\,<\,6.76$	$1.87\,<\,1.97\,<\,2.05$
$\hbox {R}^2$ charged	$0.14\,<\,0.20\,<\,0.25$	$0.46\,<\,0.53\,<\,0.59$
MUE charged	$5.50\,<\,5.67\,<\,5.84$	$1.86\,<\,2.01\,<\,2.19$

Considering the whole dataset of molecules, *model A* and *B* present the same trend and a similar statistical distribution. Comparing the $\hbox {R}^2$ and MUE to the *dominant species approximation*, *model A* and *B* show a drastic improvement with a $\hbox {R}^2 = 0.36\,<\,0.40\,<\,0.45$ and a $\hbox {MUE}=1.95\,<\,2.03\,<\,2.11$ and $1.90\,<\,1.98\,<\,2.06$ for *model A* and *B* respectively. For the protonated species, both models have a similar $\hbox {R}^2$ comparable with the *dominant species approximation*, but an improvement in MUE, going from $15.45\,<\,15.59\,<\,15.74$ to $2.00\,<\,2.11\,<\,2.24$ for *model A* and from $15.54\,<\,15.68\,<\,15.82$ to $1.95\,<\,2.06\,<\,2.18$ for *model B*. **81** is the largest outliers for these two models, with a $\log D=-8.1\pm 0.5$ and $-8.3\pm 0.6$ for *model A* and *B* respectively, while the experimentally measured data is $\log D=-2.2\pm 0.3$.

Again, charging corrections (*model C*) do not work well when applied to the whole dataset, resulting in a high $\hbox {MUE}=6.57\,<\,6.67<6.76$ and a low $\hbox {R}^2 = 0.05<0.07<0.09$. In contrast using *model D* a drastic improvement of the results is observed, resulting in a $\hbox {MUE}=1.86<2.01<2.09$ and $\hbox {R}^2 = 0.46<0.53<0.59$ for the protonated species and $\hbox {R}^2 = 0.35<0.40<0.45$ and $\hbox {MUE}=1.87<1.97<2.05$ for the entire dataset.

To test the utility of using effective pKa values in the above calculations, *model D* was compared to results obtained by application of Eq. 20 for all the charged species. For the 19 protonated molecules considered *model D* and Eq. 20 show a MUE = 2.1 and MUE = 2.3 respectively. The difference is due to 5 molecules that have different pKa and effective pKa values owing to the co-existence of multiple proto- and tautomers at pH 7.4 (**10,11, 15, 60, 63**). For these 5 molecules the *two-species approximation* performs well with a MUE = 1.0, which is significantly better than the MUE = 2.4 produced by Eq. 20. However, given the small size of the dataset, it is not possible to assert whether the improvements are statistically significant. Lastly, the relative contributions of P values for conjugate and dominant species in equation 19 were evaluated. In all cases ${P_{\mathrm{A^{\mathrm{con}}}}}\gg{P_{\mathrm{A^{\mathrm{dom}}}}}$ and the contribution of the second term on the right hand side of equation 19 could be neglected without impact on the calculated log *D* values.

Comparison of the *two-species approximation* results with other SAMPL submissions indicate significant improvements. In terms of MUE *model D* is now comparable to the top ranked submissions, and R values $(0.59<0.63<0.67)$ are in line with the best performing molecular dynamics based methods [44], though still inferior to the top-ranked submissions that used other methodologies.

### Reproducibility of results between different simulation packages

**Fig. 6:**
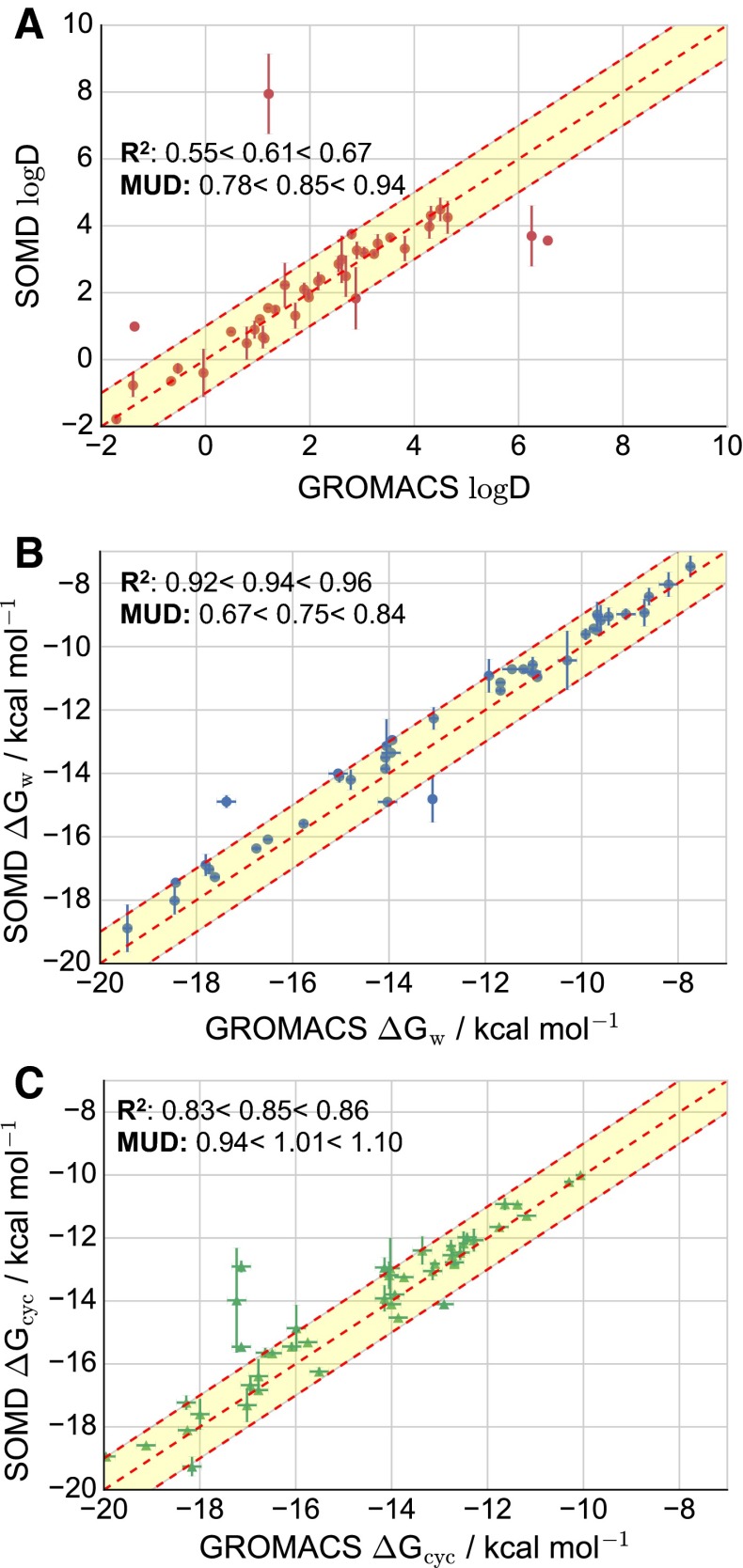
Comparison between SOMD and Gromacs $\log D$ **A** *red circles*, hydration free energy **B** *blue circles* and solvation free energy in cyclohexane **C** *green triangles*. All MUD values of solvation free energies are given in $\hbox {kcal}\cdot \hbox {mol}^{-1}$. *Dashed red line* shows perfect correlation between datasets and *shaded yellow area* a 1 $\log D$ (**A**) and 1 $\hbox {kcal}\cdot \hbox {mol}^{-1}$ (**B** and **C**) deviation bound

The consistency and reproducibility of predicted distribution coefficients were analyzed by comparing results of *model B* with those reported by the Mobley group (UCI) [44], under the same assumption that all the molecules are neutral. The same input files were used, but free energy calculations were performed with the software Gromacs [45] and results are reported in Fig. 6. The SOMD free energies Fig. 6 **b**, **c** and $\log D$ values Fig. 6 **a** are computed separately for each of the two runs. Reported values are averages of the two runs and their standard deviation according to Eq. 21. Comparing $\log {\textit D}$ predictions, a fair agreement is observed with $\hbox {R}^2 = 0.55<0.61<0.67$ and the mean unsigned deviation is MUD = $0.78<0.85<0.94\,\log D$ units. **83** is the largest outlier in the SOMD prediction with a $\log D = 7.9\pm 1.2$ while the computation with Gromacs gives $\log D = 1.21\pm 0.09$. The next outlier is molecule **17** with a SOMD $\log D = 3.7\pm 0.9$ and a Gromacs $\log D =6.25\pm 0.04$, followed by **82** SOMD $\log D=3.6\pm 0.1$ and Gromacs $\log D=6.56\pm 0.05$. Additionally, comparing solvation free energy predictions between SOMD and Gromacs, differences in cyclohexane solvation free energy for **82** and **17** are present. In particular, **82** is the largest outlier, with an absolute difference between SOMD and Gromacs predictions of 4.2 $\hbox {kcal}\cdot \hbox {mol}^{-1}$, while **17** shows a difference of 3.3 $\hbox {kcal}\cdot \hbox {mol}^{-1}$. Nonetheless, the free energy predictions are overall in better agreement, with $\hbox {R}^2 = 0.92<0.94<0.96$ and MUD = $0.67<0.75<0.84\,\hbox {kcal}\cdot \hbox {mol}^{-1}$ for hydration free energy and $\hbox {R}^2 = 0.83<0.85<0.86$ and MUD = $0.93<1.01<1.10\,\hbox {kcal}\cdot \hbox {mol}^{-1}$ for solvation free energy in cyclohexane.

In the Gromacs protocol used, alchemical free energies were computed with 20 $\lambda$ windows both for the discharging and vanishing step and also using PME [45] for electrostatic calculations. In contrast, SOMD uses nine $\lambda$ windows for the discharging step and 12 for the vanishing one, along with Barker-Watts atom based reaction field [40]. These protocol differences may be the source of variability; further investigation beyond the scope of this report is needed to clarify the origin of the discrepancies.

## Conclusions

Alchemical free energy calculations were carried out with Sire/OpenMM 6.3 (rev. 2015.0.1) [13, 14] to determine the distribution coefficient for 53 drug-like molecules in the context of SAMPL5. Overall, *model A, B, C* and *D* were not consistent with experimental values. In particular a high mean unsigned error is recorded for all models using the submitted *dominant species approximation*. A retrospective analysis of the organisers shows a Pearson $R = 0.4\pm 0.2$ for *model C* and $R=0.6\pm 0.2$ for all *models A,B*. In contrast quantum mechanical based methods such as COSMO-RS [47] fared much better than molecular mechanical approaches, where the best submission achieved an average Pearson $R=0.84\pm 0.04$ and a $\hbox {MUE}=1.7\pm 0.2\,\log \hbox {D}$ units [48]. The *two-species approximation* that was introduced after the competition had finished fares much better than the submitted result and is much closer to the top performing submissions (results from *model D* are $R=0.59<0.63<0.67$ and $\hbox {MUE}=1.87<1.97<2.05$).

Two major problems could be identified that significantly influenced the outcome of the calculations. Firstly, pKa estimations indicated that many of the SAMPL5 solutes could adopt multiple protonation states in aqueous solution at the pH at which measurements were conducted. Since this greatly complicated the number of simulations to carry out a *dominant species approximation* was made whereby only the (likely) most populated species was considered in each phase for vacuum to water/cyclohexane solvation free energy calculations. This turned out to be a poor approximation since this lead to vastly too negative $\log D$ values for ionizable molecules. In addition, rigorous evaluation of the gas phase free energy change for converting between neutral and dominant species, initially neglected, was in fact problematic because of the lack of a straightforward scheme to account for the contribution of dummy atoms. Indeed $\log D$ predictions from the Mobley lab (UCI) were generally more accurate owing to their use of a different (albeit drastic) assumption whereby all solutes were only considered to exist in aqueous or organic phases in a neutral form only [48]. Further use of the *dominant species approximation* is not recommended.

A retrospective analysis introduced a *two-species assumption* that allowed for equilibration of ionised and neutral forms of an ionisable solute in aqueous and organic phases. This model greatly reduced errors for charged molecules, bringing them in line with the results obtained for non ionisable species. The approach produced small improvements in accuracy on this dataset in comparison with the more commonly used pKa correction of log P values given by equation 20. Further inspection of the results demonstrated that the contribution of charged species $(P_{\mathrm{A}^{\mathrm{dom}}})$ to the predicted $\log D$ values was negligible. While this suggests that evaluation of vacuum to cyclohexane transfer free energies of charged species are unnecessary, it will be interesting to evaluate this assertion in more complex scenarios where for instance charged solutes partition into cyclohexane together with clusters of water molecules. The approach could be further generalised to handle more complex molecules that adopt multiple charge states, but a drawback is that the results depend on the values of ionisation and tautomerisation equilibrium constants. Consequently robust predictions will require accurate computation of vacuum to solvent transfer free energies, and also pKa constants.

A second source of error was introduced by finite size electrostatics corrections. Such correction terms are essential to yield hydration free energies of cationic species in agreement with experimental data. Results from the Minnesota dataset indicate that this correction term only has a small influence on the hydration free energy of neutral species in water. However, the effect is more pronounced when the correction term is applied to polar solutes in cyclohexane. This was done here to capture some polarisation effects since the static dielectric constant of GAFF cyclohexane is 1.0, whereas the experimental value is approximately 2.0. Unfortunately, the present attempt to add this missing physics to GAFF fails to convince, since the accuracy of $\log D$ predictions systematically worsens. A possible explanation is that the GAFF force field as used here is unbalanced and favors solvation of solutes in a non-polar solvent. Indeed, evaluation of the $\log D$ results for non-ionisable solutes where finite-size electrostatics correction terms were not applied suggests that the partitioning between water and cyclohexane is generally overly favourable for the organic phase.

In conclusions, predictions of $\log D$ values by molecular simulations proved particularly difficult in SAMPL5 owing to the need to deal with pKa corrections and with shortcomings of non-polarisable force-fields for modelling transfer between polar/non-polar solvents. For future efforts and with a view to improve the robustness of molecular simulation protocols, it would be useful to devise datasets that enable testing of these separate sources of errors. This could be done by separating datasets into compounds predicted to adopt a single protomer/tautomer form in aqueous and organic phases, and ionisable compounds that may adopt multiple charged states. In the first case, log *D* and log *P* are equivalent and their evaluation does not require pKa considerations. Ideally forcefields validated on this category of compounds could be then combined with pKa estimators to address the more challenging (albeit common) situation where multiple species contribute to a $\log D$ value.

## Electronic supplementary material

Below is the link to the electronic supplementary material.

Supplementary material 1 (pdf 771 KB)

Supplementary material 2 (zip 8 KB)

Supplementary material 3 (zip 3 KB)
