# Supplementary material for: Blinded predictions of distribution coefficients in the SAMPL5 challenge
Source: J Comput Aided Mol Des. 2016 Sep 27;30(11):1101–14. doi: 10.1007/s10822-016-9969-1 (PMC5206288; doi:10.1007/s10822-016-9969-1)
Supplement: Supplementary file 1 — Supplementary material 1 (pdf 771 KB) [file 10822_2016_9969_MOESM1_ESM.pdf]

Supplementary Information

SAMPL5: Distribution Coefficient Predictions with SOMD

S. Bosisio, A. S.J.S. Mey, J. Michel

August 23, 2016

Datasets

Fig. 1 shows the dataset extracted from the Minnesota Database [1]. The dataset was chosen in order to simulate the most typical moieties present in the SAMPL5 batch.

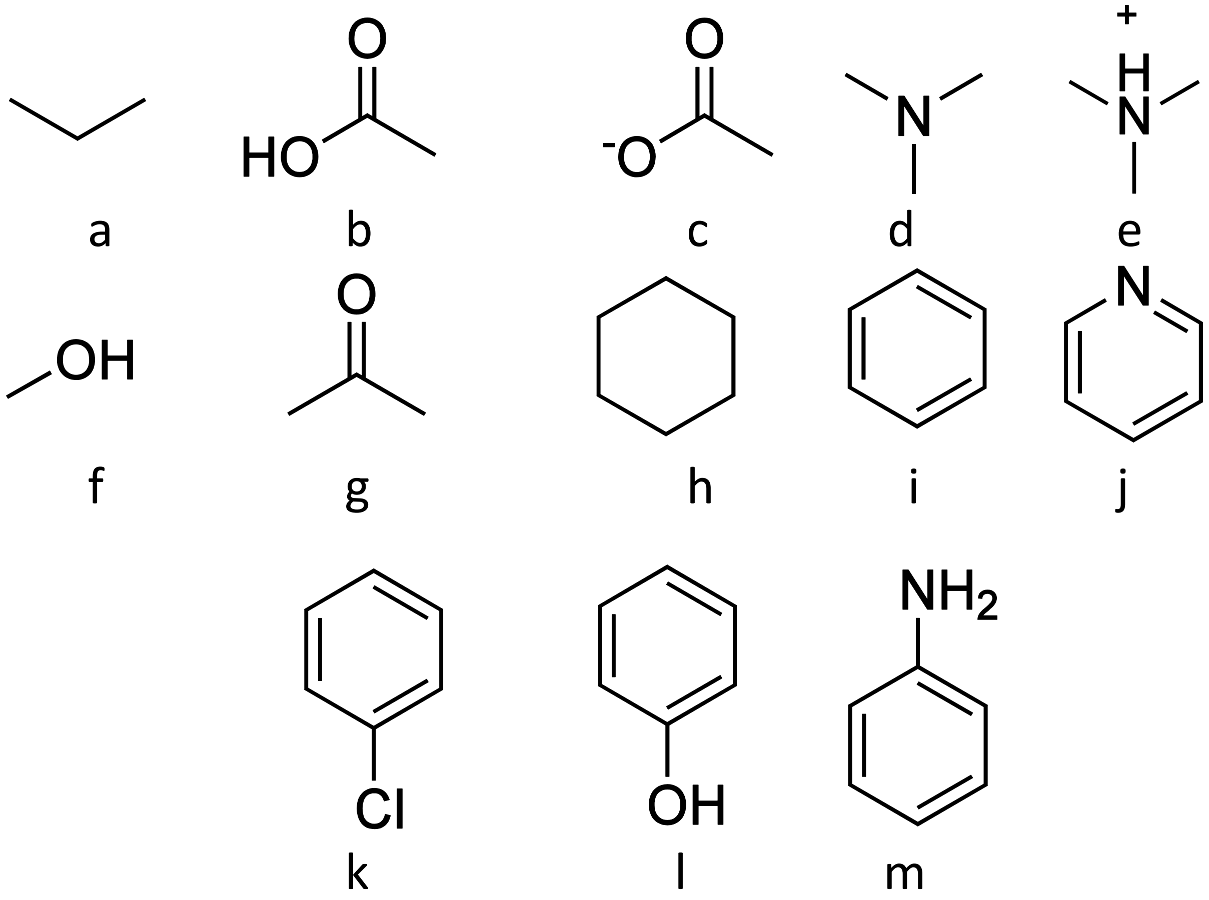

Figure 1: Dataset of molecules selected from the Minnesota Database [1]: a) n-propante, b) acetic acid c) acetate d) trimethylamine e) trimathylammonium f) methanol g) acetone h) cyclohexane i) benzene j) pyridine k) chlorobenzene l) phenol m) aniline

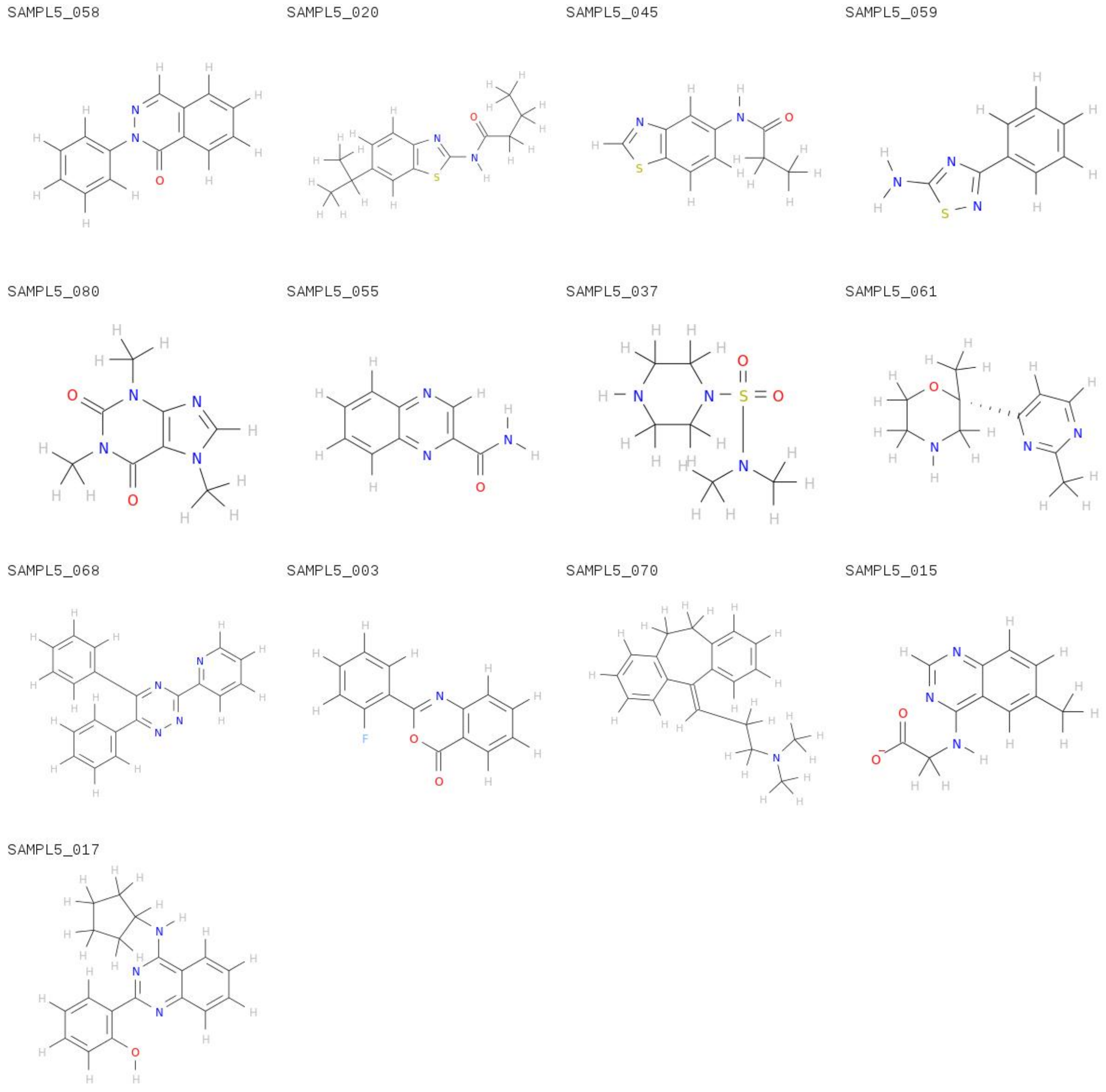

(a) SAMPL5 distribution coefficient molecules of batch 0

Fig. 2a, 2b, 2c show the 53 molecules of SAMPL5 dataset divided in batch 0, batch 1 and batch 2

SAMPL5_063

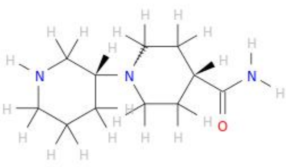

SAMPL5_071

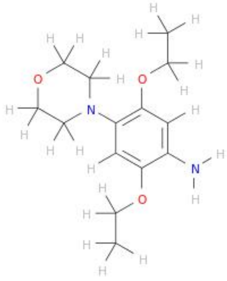

SAMPL5_072

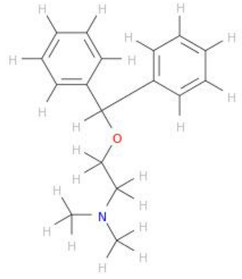

SAMPL5_011

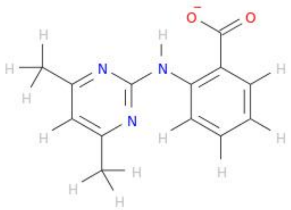

SAMPL5_027

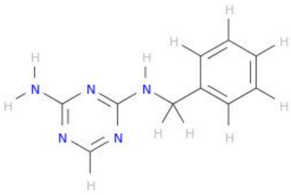

SAMPL5_056

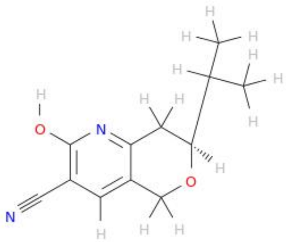

SAMPL5_047

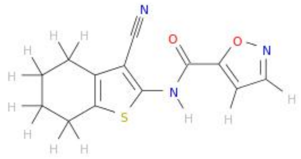

SAMPL5_005

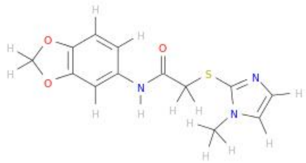

SAMPL5_090

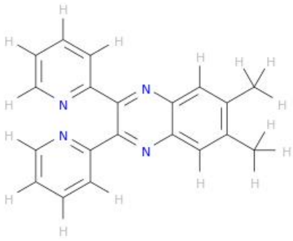

SAMPL5_021

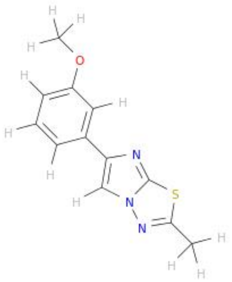

SAMPL5_004

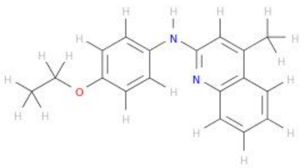

SAMPL5_081

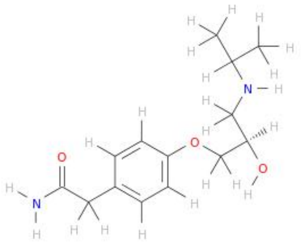

SAMPL5_007

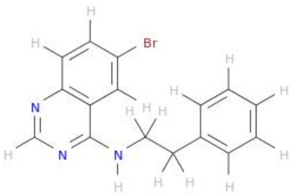

SAMPL5_042

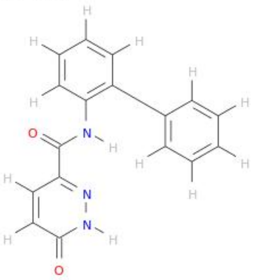

SAMPL5_010

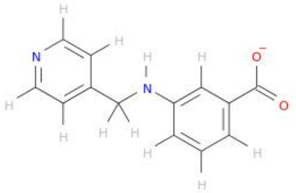

SAMPL5_048

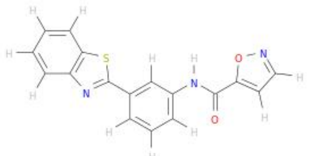

(b) SAMPL5 distribution coefficient molecules of batch 1

SAMPL5_019

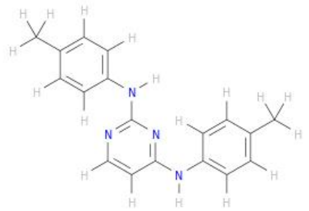

SAMPL5_086

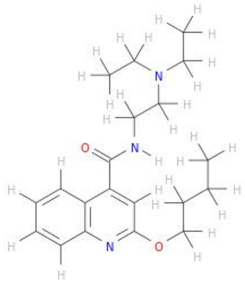

SAMPL5_049

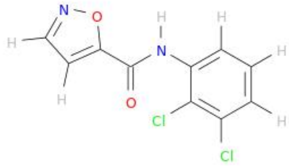

SAMPL5_088

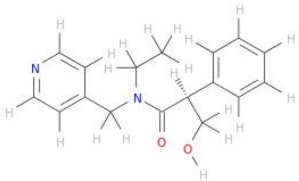

SAMPL5_024

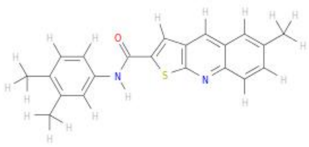

SAMPL5_074

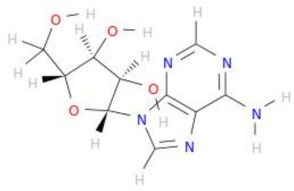

SAMPL5_050

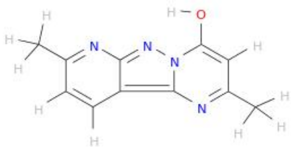

SAMPL5_085

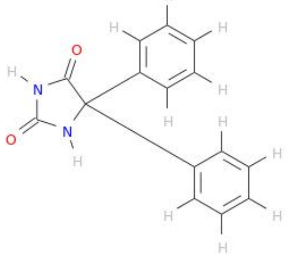

SAMPL5_067

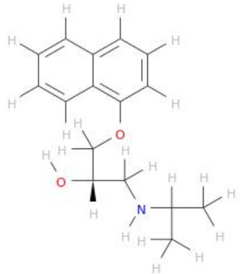

SAMPL5_069

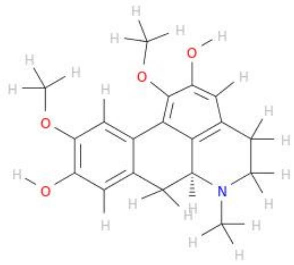

SAMPL5_013

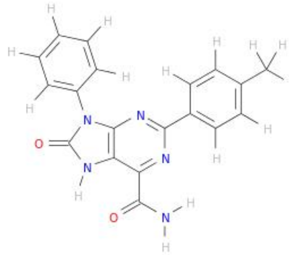

SAMPL5_092

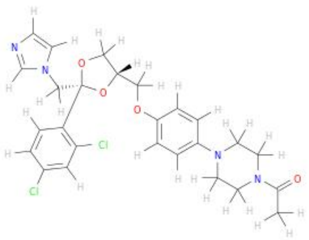

SAMPL5_083

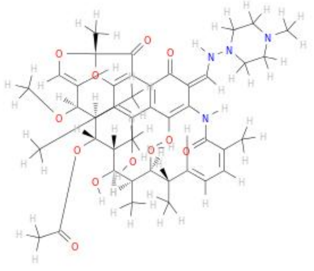

SAMPL5_075

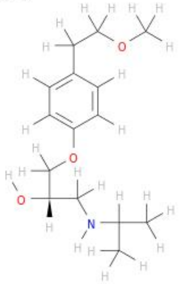

SAMPL5_002

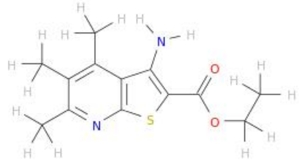

SAMPL5_084

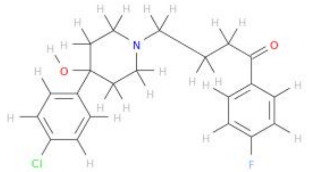

(c) SAMPL5 distribution coefficient molecules of batch 2

Chemical structures for the charged species are represented in fig. 3

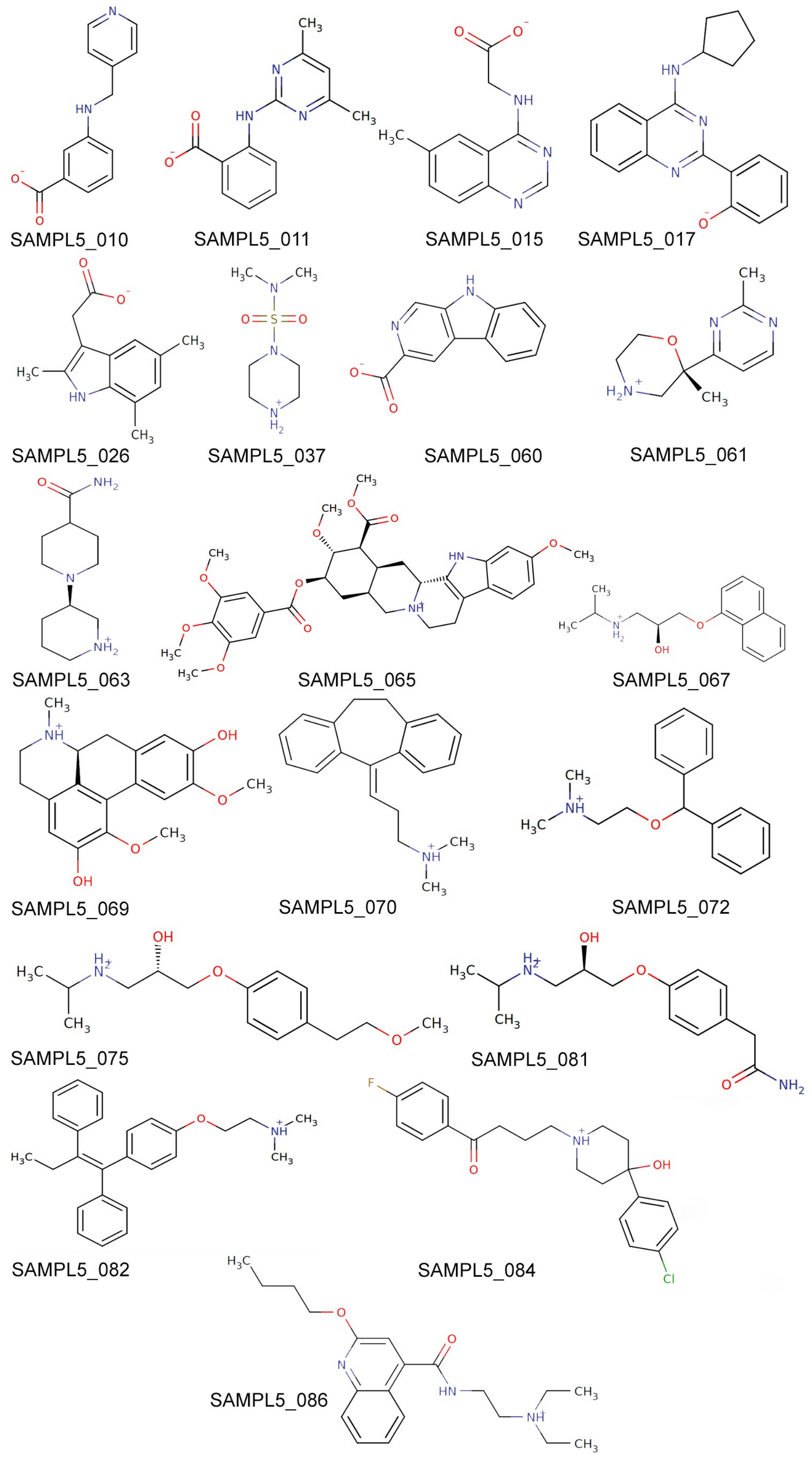

Figure 3: Chemical structure for the compounds modeled as charged

Statistical analysis of the different models

The statistical significance of *model A, B, C* and *D* of the **dominant-species model** is shown in fig. 4. Overlap betwween notches is present in *model A* and *B*, denoting a similar behavior in $\log$ D estimations. *Model C* is the less statistical significant with the lowest R^2 and τ and highest MUE.

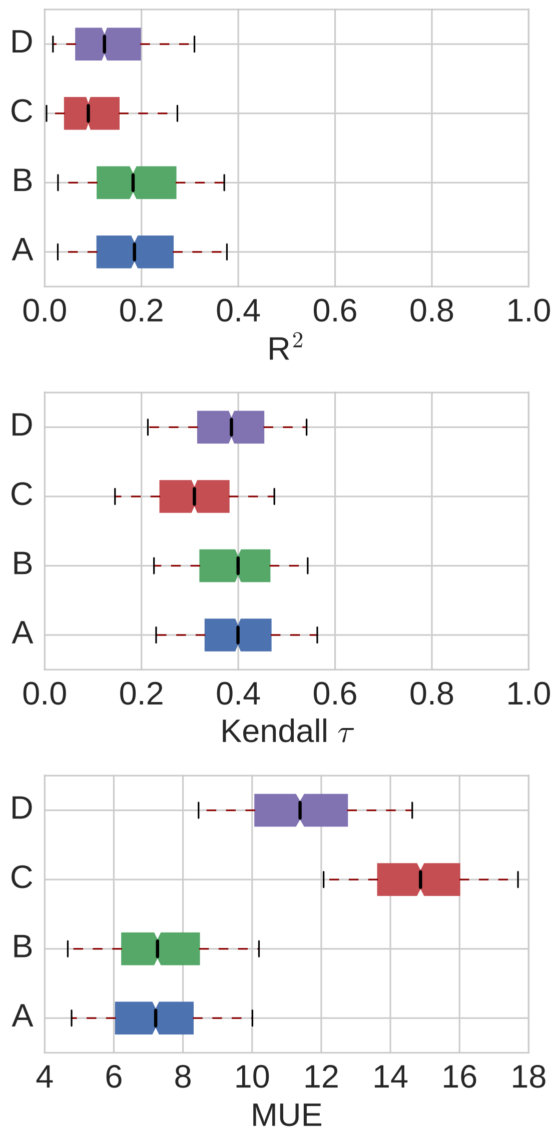

Figure 4: Comparison of determination coefficient R^2 (top), Kendall τ (middle) and mean unsigned error (MUE) (bottom), between *model A, B, C* and *D* for $\log$ D estimation with **dominant-species model**. Results show in box plot form with the 5th 95th percentile, the median and the notch.

Results are improved if the **two-species model** is adopted and statistical significance of *model A, B, C* and *D* is shown in fig. 5

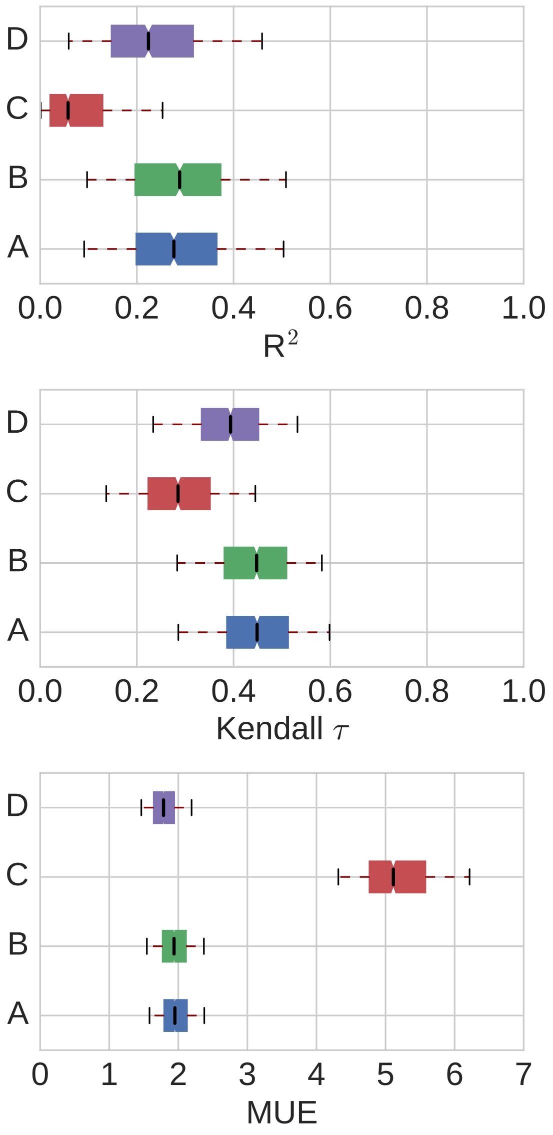

Figure 5: Comparison of determination coefficient R^2 (top), Kendall τ (middle) and mean unsigned error (MUE) (bottom), between *model A, B, C* and *D* for $\log$ D estimation with **two-species model**. Results show in box plot form with the 5th 95th percentile, the median and the notch.

The archive dominant_species.zip contains:

- logD folder: the predicted $\log$ D are stored into csv files along with the standard error for *model A* (A.csv), *B* (B.csv), *C* (C.csv) and *D* (D.csv)
- solv_energies folder: this folder contains the solvation free energies for each model and standard error for cyclohexane (cyclohexane_solv.csv) and hydration free energies (water_solv.csv)

The archive two_species.zip contains:

- Comparison_methods.csv : a comparison between two species equation (eq.19) and eq.20 [2] to test the efficacy of the effective pKa assumption
- Concentration.csv: the concentration used to calculate the $\log$ D with eq.19, retrieved from ChemAxon [3]
- logD_charged.csv: the predicted $\log$ D for each charged species according to *model A, B, C* and *D* for the two species approach
- pKa.csv: the pKa used to calcualte the $\log$ D with eq.19, retrieved from ChemAxon [3]

References

[1] Aleksandr V. Marenich, Casey P. Kelly, Jason D. Thompson, Gregory D. Hawkins, Candee C. Chambers, David J. Giesen, Paul Winget, Christopher J. Cramer, and Donald G. Truhlar. *University of Minnesota, Minneapolis*, 2009.

[2] Robert A. Scherrer, Susan M. Howard, *J. Med. Chem.*, 1977, 20(1), 53-58

[3] Chemaxon, www.chemicalize.org.
